# The effects of perceived social support and psychological capital on attitudes towards professional psychological help-seeking among Chinese college students: The mediating role of psychological help-seeking stigma

**DOI:** 10.1371/journal.pone.0344735

**Published:** 2026-03-12

**Authors:** YingYing Xie, LiHua Yao, ChengRui Ye

**Affiliations:** School of Public Health, Chongqing Medical University, Chongqing, China; University of Tabuk, SAUDI ARABIA

## Abstract

**Objective:**

This study grounded in resource conservation theory, examines the association between perceived social support and psychological capital on college students’ attitudes toward seeking professional psychological help, with particular emphasis on the mediating role of stigma for seeking professional psychological help. The aim is to provide a reference framework for promoting mental health among college students.

**Method:**

We employed a cross-sectional design, investigated perceived social support, psychological capital, stigma for seeking professional psychological help, and attitudes toward seeking professional psychological help among 3,012 Chinese college students.

**Result:**

The results indicated that perceived social support, psychological capital, and attitudes toward seeking professional psychological help were negatively associated with the stigma for seeking professional psychological help. Perceived social support and psychological capital were directly and positively associated with attitudes toward seeking professional psychological help. Furthermore, the stigma for seeking professional psychological help was found to mediate the association between perceived social support and psychological capital on attitudes toward seeking professional psychological help (*β* = 0.1 [0.059, 0.138], *β* = 0.248 [0.21, 0.29]). This mediation explained 16% and 39% of the total association.

**Conclusion:**

This study highlights the association between perceived social support and psychological capital and attitudes toward seeking professional psychological help among college students, while also identifying mediating role of stigma for seeking professional psychological help. These findings provide empirical support for designing effective interventions to improve college students’ attitudes toward seeking professional psychological help.

## Introduction

Mental health and psychiatric care are fundamental pillars of public health. Nevertheless, driven by rapid socioeconomic development, the tempo of modern life has markedly accelerated, and psychological stressors have steadily increased, leading to a global surge in mental health disorders [1–3]. Imposing both individual and societal burdens, mental illness profoundly impacts individuals’ daily lives from psychological and behavioural perspectives. In this context, seeking professional psychological support becomes increasingly indispensable.

Attitudes toward seeking professional psychological help refer to an individual’s inherent predisposition to seek assistance from professionals when experiencing psychological or emotional distress, and to address such distress through professional advice, counseling, and treatment [4]. This attitude constitutes a significant manifestation of mental health coping strategies and serves as a predictor of whether individuals will seek professional psychological help. However, research indicates that most college students still exhibit a passive attitude when confronted with psychological distress, failing to fully utilize existing psychological support resources. A multinational study revealed that many college students refrain from seeking assistance due to embarrassment, preferring to resolve issues independently. Only 24.6% of college students indicated that they would seek psychological support if emotional difficulties arise [5]. A separate study on psychological distress and help-seeking revealed similar patterns: among British college students experiencing psychological distress, fewer than one in three were willing to accept formal psychological support [6]. Research among American college students indicated that, among those suffering from depression or anxiety, the proportion not receiving any services ranged from 37% to 84%. The proportion of students willing to seek psychological help was far lower than the proportion experiencing psychological difficulties [7–8]. These data reflect the prevailing reluctance among college students to seek professional psychological assistance. When facing psychological distress, Chinese students often exhibit an even more negative attitude towards seeking mental health support [9]. They often equate seeking professional psychological support with admitting mental health issues, experiencing self-doubt and societal rejection, and fearing stigmatization as ‘pathological.’ This leads individuals to conceal their psychological distress and avoid therapy [10]. When seeking professional psychological help carries significant stigma, it may reduce individuals’ willingness to access such support [11].

The stigma for seeking professional psychological help refers to the negative societal stereotypes attached to individuals or groups who seek such assistance [12]. Such stereotypes often manifest as prejudices and discrimination, fostering social alienation and exclusion. These external biases exacerbate the stigma experienced by patients, triggering feelings of shame and resistance to seeking professional psychological support. However, when individuals encounter external pressures, if they can promptly perceive understanding and support from others, which may help them gain psychological security amidst adversity and gradually transform this external assistance into stable, reliable internal resources [13].

Perceived social support refers to an individual’s sense of the various forms of support available within society [13]. This perception holds significant relevance to an individual’s mental health, serving to enhance functioning and buffer against the impact of adverse life events, thereby improving psychological health [14]. Moreover, it is recognized that perceived social support is closely associated with heightened levels of well-being, life satisfaction and quality of life, and can alleviate feelings of loneliness, depression and anxiety [15–18].

Psychological capital refers to a positive psychological state exhibited by individuals during their growth and development. It constitutes an intrinsic positive psychological quality enabling individuals to cope with change and pressure, and is composed of multiple positive psychological resources. This reflects the positive resources possessed by the individual [19]. When higher psychological capital is present, individuals may exhibit more positive cognitive attitudes and stronger adaptive capacities when confronting challenges [20]. They are better equipped to engage self-regulation in a timely manner, and when faced with psychological difficulties, are more inclined to adopt proactive coping strategies rather than resorting to avoidance or denial.

College students’ attitudes toward seeking professional psychological help can influence whether they engage in such help-seeking behaviour. Improving and promoting these attitudes is a crucial step in enhancing their mental health. Current research primarily focuses on factors such as mental health literacy, personality traits and suicidal ideation influencing attitudes toward seeking professional psychological help [21–23]. However, there remains a lack of systematic and in-depth empirical research on how perceived social support and psychological capital are associated with attitudes toward seeking professional psychological help, and what role stigma plays in this context. Therefore, grounded in resource conservation theory, this study aims to systematically elucidate the pathways through which perceived social support and psychological capital are associated with attitudes toward seeking professional psychological help, focusing on how stigma acts upon psychological help-seeking. This research enriches the existing literature on attitudes toward seeking professional psychological help and provides crucial data support for designing precise intervention strategies, offering empirical support for promoting college students’ attitudes toward seeking professional psychological help.

### Theoretical framework

Resource Conservation Theory (COR), proposed by Stevan E. Hobfoll, posits that individuals possess an intrinsic motivation to maintain, protect, and acquire crucial resources within environmental interactions [24]. Resources constitute the core elements individuals employ to evaluate and respond to stressful situations, including material possessions, energy reserves, personal characteristics, and social conditions. COR emphasizes that the level of resource conservation directly influences an individual’s stress adaptation mechanisms: those with abundant resources tend to adopt proactive coping strategies, whereas those with scarce resources are more prone to stress reactions and psychological exhaustion. Moreover, the principle of loss aversion posits that individuals are significantly more sensitivity to resource losses than to anticipated resource gains. Consequently, safeguarding existing resources from erosion constitutes the primary motivator for individual behavioral responses. Additionally, resource investment constitutes a dynamic process. When individuals experience resource scarcity, they become more susceptible to resource depletion, further diminishing their capacity to acquire new resources, thereby creating a loss spiral. Conversely, when individuals possess resource utilization capabilities, resource investment can generate new opportunities for resource acquisition, thus achieving a gain spiral of resource accumulation [25]. Under stressful conditions, resource investment not only mitigates the negative effects of resource depletion but may also foster adaptive growth and psychological development, thereby establishing a positive resource-building cycle.

Based on resource conservation theory, this study conceptualizes perceived social support and psychological capital as positive psychological resources, while attitudes toward seeking professional psychological help represent decision outcomes grounded in resource cost-benefit assessments. Perceived social support and psychological capital serve as pivotal personal resources capable of effectively mitigating the impact of stigma for seeking professional psychological help. High levels of perceived social support provide resource buffering, reducing perceived threats such as self-denial and reputational loss potentially arising from help-seeking behaviors, while individuals with high psychological capital are better able to harness positive psychological resources such as self-efficacy and optimism, reframing stigma as a manageable challenge rather than a threat. The stigma for seeking professional psychological help functions as a depleted resource within this process. When stigma is pronounced, individuals perceive that seeking assistance will result in the loss of resources such as social reputation and self-esteem. This anticipation of significant loss fosters a negative attitude towards seeking professional psychological help. In contrast, individuals with high perceived social support and psychological capital can mitigate stigma threats, cultivating a positive attitude. Successful help-seeking is positively related to mental health, thereby further expanding their resource pool and creating a virtuous cycle. Conversely, individuals with low perceived social support and psychological capital experience greater resource depletion and heightened stigma threat. They develop negative attitudes that lead to avoidance of help-seeking. This results in the continuous depletion of psychological resources, trapping them in a loss spiral. Therefore, enriching individuals’ reserves of resources for perceived social support and psychological capital, aiming to altering the stigma for seeking professional psychological help, is a crucial pathway to enhancing attitudes toward seeking professional psychological help.

### Perceived social support and attitudes toward seeking professional psychological help

Previous research indicates that perceived social support not only serves as a potential protective factor against mental disorders but also alleviates anxiety during the help-seeking process [18]. When individuals perceive respect, understanding, and encouragement from others, they subconsciously interpret this as acceptance rather than judgement by the group. This sense of validation from others can elevate one’s self-esteem and alleviate feelings of helplessness and shame when confronting psychological distress [26]. Therefore, those who perceive stronger social support tend to harbor greater hope for the future. Bolstered by this positive perception of social support, they demonstrate greater courage in overcoming adversity and are more inclined to trust professionals and accept assistance from others [27].

Therefore, the hypotheses of this study were formulated:

H1: Perceived social support is positively associated with attitudes toward seeking professional psychological help.

### Psychological capital and attitudes toward seeking professional psychological help

Research has revealed that individuals with higher psychological resilience demonstrate greater self-awareness and attentiveness to their own mental health, enabling them to recognize subtle shifts in their psychological state [28]. When confronted with significant stress, they proactively seek professional support, demonstrating a strong demand for mental health services. Furthermore, those possessing greater psychological capital typically exhibit heightened self-confidence and a more optimistic outlook. Psychological capital is significantly associated with alleviating psychological distress [29]. When facing pressure and adversity, they display superior self-control and adaptability, effectively mitigating the negative impact of distressing emotions they have greater confidence and courage in expressing their feelings and concerns [30–31]. Individuals possessing ample psychological resources not only maintain a positive and optimistic attitude towards seeking professional psychological help but also have clear objectives, viewing such help as a means to achieve their goals. This effectively stimulates the motivation to seek professional psychological help when confronting psychological distress [32].

Therefore, the hypotheses of this study were formulated:

H2: Psychological capital is positively associated with attitudes toward seeking professional psychological help.

### The mediating role of the stigma for seeking professional psychological help

Due to social prejudice and discrimination against mental illness, and high public stigma, people with mental illness often experience a strong sense of shame and stigma regarding seeking psychological help [33]. College students, as a social group, are inevitably be affected by it. However, a high level of perceived social support can enhance college students’ subjective perception of social support resources, generating more positive emotions. This, in turn, reduces their negative evaluations of mental illness and helps them cope with it more positively [34]. While perceived social support may buffer the impact of discrimination, low levels of perceived social support can exacerbate these feelings [35]. Moreover, existing research has revealed that social prejudice, discrimination, and the marginalization of individuals experiencing mental health issues contribute to the stigmatization of mental illness and seeking help among college students, thereby reducing the likelihood of seeking professional psychological support [36–37].

Research on the stigma for seeking professional psychological help and psychological capital reveals that when confronted with mental health issues, individuals possessing higher psychological capital develop greater self-confidence and self-efficacy [38]. They are better equipped to self-regulate and manage their emotions, approaching adversity with a hopeful and optimistic mindset, thereby mitigating the stigmatizing effects of seeking psychological support [32]. Conversely, individuals with lower psychological capital face greater challenges to their self-esteem, resilience, and sense of self-efficacy, leading to self-doubt [39]. This exacerbates rumination and symptoms of mental health issues among college students, while simultaneously deepening the stigma associated with seeking professional psychological help [40]. Such individuals tend to harbor pessimistic expectations about their present circumstances or future prospects, rendering them even more reluctant to seek professional psychological help.

In summary, if an individual possesses insufficient psychological resources and a lack of perceived social support and psychological capital, they will be unable to avoid or prevent psychological attrition. This may exacerbate the stigma for seeking professional psychological help, ultimately leading to a negative attitude towards seeking professional psychological help and avoidance of it. This is detrimental to the positive resolution of psychological issues among college students.

**Fig 1:**
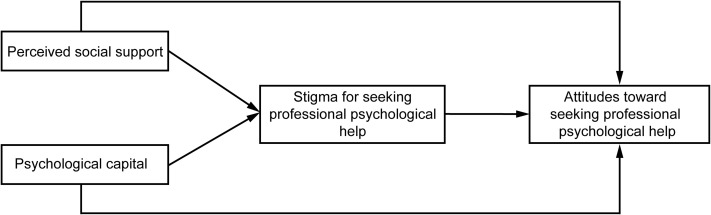
Hypothesized model diagram for perceived social support, psychological capital, and stigma for seeking professional psychological help in attitudes toward seeking professional psychological help.

Therefore, the hypotheses of this study were formulated as shown in Fig 1:

H3: Stigma for seeking professional psychological help is a mediator in the association between perceived social support and attitudes toward seeking professional psychological help.

H4: Stigma for seeking professional psychological help is a mediator in the association between psychological capital and attitudes toward seeking professional psychological help.

## Materials and methods

### Participants

This study employed a questionnaire survey methodology. Constrained by time, resources and access limitations, convenience sampling was used to recruit college students for participation. However, we consciously recruited participants from diverse faculties to enhance internal sample heterogeneity. Throughout the survey, we emphasized the complete anonymity of the questionnaire and the confidentiality of data, which would be used solely for academic research purposes. This approach aimed to minimize participation avoidance stemming from concerns over privacy and perceived evaluation.

A total of 3,450 samples were collected, with 3,012 included in this study, yielding an effective response rate of 87.3%. Among them, 995 (33%) are male and 2,017 (67%) are female; 2,503 (83.1%) are < 20 years old, 509 (16.9%) are 20–24 years old; 1,035 (34.4%) are freshmen, 774 (25.7%) are sophomores, 634 (21%) are juniors, 569 (18.9%) are seniors; 1,390 (46.1%) are rural students and 1,622 (53.9%) are urban students; 957 (31.8%) are only children and 2,055 (68.2%) are non-only children; 746 (24.8%) are classroom cadres and 2,266 (75.2%) are non-classroom cadres. The recruitment period for this study commenced on 1 May 2025 and concluded on 1 September 2025, strictly adhering to the Declaration of Helsinki and complying with relevant institutional and national guidelines. All participants provided informed consent, with the document reviewed and approved by the Medical Ethics Committee of Chongqing Medical University (Approval No.: 2025−040). The study exclusively involved adult participants, with no minors included.

### Measures

#### Perceived social support scale (PSSS).

The Perceived Social Support Scale was originally developed by Zimet et al. [41]. Then, Qianjin Jiang undertook a systematic Chinese adaptation of Zimet’s Perceived Social Support Scale. This involved a process of expert forward translation, followed by back-translation by other specialists, cultural adaptation, and finally testing the scale’s reliability and validity. The revised version confirmed the validity of the original three-factor structure among Chinese populations and established sound reliability and validity indices [42]. The scale consists of 12 self-assessment items and has three parts: family support, friend support and other support. It’s primarily conducted through self-evaluation. The scale is based on a 7-point Likert scale, with a higher total score indicating a stronger sense of social support and greater satisfaction with it. In this study, the Cronbach’s α coefficient for the total scale was 0.95, which is higher than 0.7, indicating that the Perceived Social Support Scale has good internal consistency.

#### Psychological capital questionnaire (PCQ).

The Psychological Capital Questionnaire was originally developed by Luthans et al. [43]. Based on previous research Kuo Zhang and colleagues drew upon Luthans et al.’s psychological capital questionnaire as a blueprint, using a Chinese-translated version that had undergone preliminary domestic use as their foundation. They first optimized the structure through item analysis and exploratory factor analysis, removing items with poor factor loadings. Subsequently, confirmatory factor analysis was employed to test the structural validity of a four-factor model. Ultimately, validation with college students confirmed the revised scale’s sound reliability, establishing its suitability for local application [44]. Psychological Capital Questionnaire consisted of 26 questions, including four dimensions of self-efficacy, optimism, resilience, and hope. The assessment is primarily conducted through self-evaluation. A 7-point Likert scale was used, and the sum of the ratings of each topic was the total score of the questionnaire. Higher total scores indicate higher levels of positive psychological capital. In this study, the Cronbach’s α coefficient for the total scale was 0.94, which is higher than 0.7. This indicates that the Psychological Capital Questionnaire exhibits good internal consistency.

#### Questionnaire of stigma for seeking professional psychological help (SSPPH).

The Stigma Scale for Receiving Psychological Help (SSRPH) and the Self-Stigma of Seeking Help Scale (SSOSH) were originally proposed by Komiya and Vogel [45–46]. Based on these scales, Zhihong Hao ensured linguistic equivalence through translation-back-translation and cultural adaptation. Cultural and content validity were ensured through expert review and cognitive interviews. Ultimately, through factor analysis and reliability-validity testing on a large-scale sample, the Chinese version of the questionnaire was validated for structural stability. Its measurement validity was also confirmed among Chinese college students [47]. The Questionnaire of Stigma for Seeking Professional Psychological Help consists of 10 entries, with 5 each for self-stigma and public stigma. The assessment is primarily conducted through self-evaluation. A 5-point Likert scale was used, with higher scores indicating that individuals perceived a higher level of stigmatization when seeking professional psychological help. In this study, the Cronbach’s α coefficient for the total scale was 0.88, which is higher than 0.7. This indicates that the Questionnaire of Stigma for Seeking Professional Psychological Help exhibits good internal consistency.

#### Attitudes toward seeking professional psychological help scale (ATSPPH).

The Attitudes toward Seeking Professional Psychological Help (ATSPPH) was originally developed by Fischer [48]. Building on this foundation, Zhihong Hao adapted the scale to Chinese cultural contexts and linguistic conventions. The original questionnaire was first translated and back-translated by postgraduate students specializing in psychology and English. Subsequently, psychological experts validated the questionnaire, culminating in the finalization of the Chinese version. The reliability and validity of this scale were verified among college students [49]. The Attitudes Toward Seeking Professional Psychological Help consists of 29 questions, including four dimensions of confidence in mental health professionals, tolerance of stigma, interpersonal openness and self-perceptions. The assessment is conducted through self-evaluation. A 5-point Likert scale was adopted, with higher scores indicating more positive attitudes toward seeking professional psychological help. In this study, the Cronbach’s α coefficient for the total scale was 0.88, which is higher than 0.7. This indicates that the Attitudes Toward Seeking Professional Psychological Help Scale exhibits good internal consistency.

### Statistical methods

This study employed Wenjuanxing for questionnaire distribution and data collection. As the online platform required participants to complete all items before submission, there was no missing data. The data were subsequently screened, and invalid responses were excluded. Invalid responses were defined as those with excessively short completion times, consecutive repeated selections, or extreme options.

This study primarily employed SPSS 27.0 for statistical data processing. First, a normality test was conducted on the data. The results indicated that the absolute values of skewness and kurtosis for all observed indicators were less than 1, and the data points on the Q-Q plot were broadly distributed along the diagonal line. This suggests that the data in this study approximates a normal distribution [50]. Second, we generally consider that when significance is absent, it indicates homogeneity of variances [51]. In this study, Levene’s test revealed no significant differences in variances between certain groups in comparisons involving attitudes toward seeking professional psychological help (e.g., gender, age, whether or not you are a class cadre, *p* = 0.42–0.998), thus satisfying the assumption of homogeneity of variances. For comparisons where this assumption was not met (e.g., grade, place of origin of students, whether or not you are an only child, *p* < 0.05), we employed results adjusted for heteroscedasticity. Variance inflation factors (VIFs) and tolerance statistics were employed to verify whether potential multicollinearity existed among variables. If a predictor variable in the model exhibited a VIF < 5 and tolerance > 0.1, this indicated the absence of multicollinearity [52]. Subsequently, as all research variables were measured via self-report questionnaires, this may have resulted in common method bias. To address this issue, we employed Harman’s single-factor method and a two-factor model to examine common method bias. All survey items were loaded onto unrotated factors, and the proportion of variance explained by the largest factor was assessed against a 40% critical threshold. Furthermore, this study incorporated a latent method factor into a four-factor model to test whether model fit indices improved significantly upon its inclusion. If no significant improvement occurs, it would indicate the absence of severe common method bias [53]. This helps ensures that common methodological biases are minimized and do not interfere with subsequent analyses. We employed descriptive statistics to express the fundamental characteristics of data. Independent samples t-tests and ANOVAs of variance were employed to examine demographic differences in attitudes toward seeking professional psychological help. Pearson correlation analysis was employed to examine the strength and direction of relationships between perceived social support, psychological capital, stigma for seeking professional psychological help, and attitudes toward seeking professional psychological help. *p* < 0.05 was considered statistically significant.

Finally, we employed item parcels to bundle perceived social support, psychological capital, and attitudes toward seeking professional psychological help, packaging each dimension into observed indicators of latent variables. This approach simplifies the model and enhances the robustness of parameter estimation [54–55]. Using Amos 24.0, a structural equation model was established in which perceived social support and psychological capital served as independent variables, stigma for seeking professional psychological help acted as the mediating variable, and attitudes toward seeking professional psychological help were dependent variable. The model was adjusted based on the correction indices, resulting in significant improvement and an acceptable model fit (*x*^*2*^*/df* = 13.515, RMSEA = 0.064, SRMR = 0.048, NFI = 0.91, IFI = 0.91, CFI = 0.91). In data analysis, we incorporated potential confounding variables arising from sampling bias as covariates within the model for control purposes. All observed variables in this study were treated as continuous variables and estimated using maximum likelihood estimation (ML). Concurrently, model estimation and hypothesis testing were conducted via 5000 repeated samples using the Bollen-Stine Bootstrap method to assess direct and mediating effects of hypotheses. Bias-corrected 95% confidence intervals were reported to enhance the robustness of results.

## Results

### Multicollinearity test

In this study, each independent variable exhibits a Variance Inflation Factor (VIF) below 5 and a tolerance exceeding 0.1. Consequently, we may conclude that multicollinearity does not pose a significant issue within this research. See Table 1 for details.

**Table 1:** Tolerance and VIF for multiple collinearity in statistical analyses of perceived social support, psychological capital, and stigma for seeking professional psychological help on attitudes toward seeking professional psychological help.

Independent variable	*B*	*β*	*t*	*p*	Tolerance	VIF
Percieved social support	0.21	0.2	11.695	< 0.01	0.61	1.638
Stigma for seeking professional psychological help	−1.072	−0.528	−34.088	< 0.01	0.749	1.335
Psychological capital	0.043	0.07	3.889	< 0.01	0.553	1.808

### Common method bias test

According to Harman’s single-factor test showed that unrotated 12 factors with eigenroots greater than 1 were obtained. The first of these factors explained 12.95% of the variance, which was less than the critical criterion of 40%.

This study further employed a two-factor model for testing, incorporating an additional latent method factor into the four-factor model. Following the inclusion of this factor, the model fit indices (*χ²/df* = 17.496, RMSEA = 0.074, SRMR = 0.042, NFI = 0.928, RFI = 0.907, IFI = 0.932, TLI = 0.911, CFI = 0.932) compared to the original four-factor model (*χ²/df* = 17.43, RMSEA = 0.074, SRMR = 0.055, NFI = 0.921, RFI = 0.907, IFI = 0.925, TLI = 0.912, CFI = 0.925) did not show any significant improvement. Therefore, it can be concluded that there was no significant common method bias in this study.

### Confirmatory factor analysis

All scales employed in this study were locally validated and are well-established instruments in widespread use. Given the substantial number of items in certain scales in this study, to enhance model parsimony and parameter the stability of parameter estimation, we applied a parceling approach during the CFA process for perceived social support, psychological capital, and attitudes toward seeking professional psychological help. This was undertaken by dimension, following the principle of content matching whereby items measuring the same dimension were grouped together. The results indicate that the factor loadings across each dimension of the model range from 0.51 to 0.97, the average common factor (AVE) values range from 0.5 to 0.75, and the composite reliability (CR) coefficients range from 0.84 to 0.9. This demonstrates that the model possesses good convergent validity and composite reliability [56].

AMOS was used to conduct a confirmatory factor analysis of the four-factor, three-factor, two-factor, and one-factor models, with RMSEA, NFI, and RFI used to assess the model fit. Although the chi-square value indicates how well the sample data fit the model, it is sensitive to the sample size, and a very large sample can easily inflate the chi-square value [57]. The sample size of this study is as large as 3,012, so other indicators need to be considered for judgment. Analysis of the results shows that the fit indices for the four-factor model are all at desirable levels and significantly better than those for the other models (RMSEA = 0.074, SRMR = 0.055, NFI = 0.921, CFI = 0.925). This suggests significant discriminant validity among perceived social support, psychological capital, psychological help-seeking stigma, and professional psychological help-seeking attitudes. See Tables 2–3 for details.

**Table 2:** Validation of scale reliability and validity indicators.

Latent Variable	Dimension	Standardized factor loading	S.E.	C.R.	*p*	CR	AVE
Perceived social support	other support	0.97				0.9	0.75
	family support	0.75	0.018	51.26	< 0.001		
	friend support	0.86	0.014	63.55	< 0.001		
Psychological capital	self-efficacy	0.91				0.9	0.7
	resilience	0.8	0.015	59.24	< 0.001		
	hope	0.8	0.015	53.34	< 0.001		
	optimism	0.81	0.015	54.08	< 0.001		
Attitudes toward seeking professional psychological help	confidence in mental health professionals	0.82				0.84	0.57
	tolerance of stigma	0.7	0.014	38.2	< 0.001		
	self-perceptions	0.8	0.02	42.93	< 0.001		
	interpersonal openness	0.69	0.018	35.44	< 0.001		
Stigma for seeking professional psychological help	self-stigma	0.58				0.9	0.5
		0.61	0.041	27.83	< 0.001		
		0.66	0.037	28.996	< 0.001		
		0.51	0.043	23.89	< 0.001		
		0.78	0.033	31.91	< 0.001		
	public stigma	0.8	0.033	31.38	< 0.001		
		0.81	0.033	31.5	< 0.001		
		0.74	0.036	29.95	< 0.001		
		0.56	0.04	25.11	< 0.001		
		0.6	0.037	26.28	< 0.001		

**Table 3:** Results of confirmatory factor analysis.

Model	*x* ^*2*^ */df*	RMSEA	SRMR	NFI	RFI	IFI	TLI	CFI
Four-factor model	17.43	0.074	0.055	0.921	0.907	0.925	0.912	0.925
Three-factor model	23.883	0.087	0.062	0.89	0.872	0.894	0.877	0.894
Two-factor model	46.302	0.123	0.098	0.784	0.753	0.788	0.757	0.788
One-factor model	66.809	0.148	0.11	0.687	0.643	0.691	0.647	0.69

Four-factor model: perceived social support, psychological capital, stigma for seeking professional psychological help, attitudes toward seeking professional psychological help;

Three-factor model: perceived social support, psychological capital, stigma for seeking professional psychological help + attitudes toward seeking professional psychological help;

Two-factor model: perceived social support, psychological capital + stigma for seeking professional psychological help + attitudes toward seeking professional psychological help;

One-factor model: perceived social support + psychological capital + stigma for seeking professional psychological help + attitudes toward seeking professional psychological help.

### Descriptive statistical analysis and demographic difference tests

In this study, the score of perceived social support was 64.44 ± 13.25; the score of psychological capital was 123.59 ± 22.75; the score of stigma for seeking professional psychological help was 19.95 ± 6.84; and the score of attitudes toward seeking professional psychological help was 105.44 ± 13.90. The specific results are shown in Table 4.

**Table 4:** Scores for each variable.

Item	overall average score	
	*x*	*s*
Perceived social support	64.44	13.25
other support	21.83	4.60
family support	20.83	5.39
friend support	21.79	4.64
Psychological capital	123.59	22.75
self-efficacy	31.97	6.79
resilience	30.46	6.75
hope	30.93	6.05
optimism	30.23	6.33
Stigma for seeking professional psychological help	19.95	6.84
self-stigma	11.03	4.07
public stigma	8.92	3.59
Attitudes toward seeking professional psychological help	105.44	13.90
confidence in mental health professionals	32.94	5.14
tolerance of stigma	20.41	3.14
self-perceptions	27.49	4.58
interpersonal openness	24.60	3.99

This study employed independent samples t-tests to examine differences in college students’ attitudes toward seeking professional psychological help across various demographic factors: gender, age, whether or not you are an only child, whether or not you are a class cadre, and place of origin. Additionally, ANOVAs were conducted to analyse the attitudes toward seeking professional psychological help among students of different grades.

The results showed that there were significant differences in the level of attitudes toward seeking professional psychological help among college students of different genders, ages, grades, place of origin, whether you are an only children, and whether you are a class cadre. Among them, female students have a higher level of attitudes toward seeking professional psychological help than male students; college students under 20 years old and freshmen have the highest level of attitudes toward seeking professional psychological help; students from cities and towns have a higher level of attitudes toward seeking professional psychological help than students from countryside; and college students who are only children and class cadres have a higher level of attitudes toward seeking professional psychological help. The specific results are shown in Table 5.

**Table 5:** Testing for differences in demographic variables regarding attitudes toward seeking professional psychological help.

Demographic variable	n	Percentage (%)	Score	*t/F*	*p*	Cohen’s *d/η²* [95% CI]
Gender				−8.53	< 0.01	−0.331 [−0.407, −0.254]
men	995	33	102.4 ± 14.05			
female	2017	67	106.94 ± 13.57			
Age				4.23	< 0.01	0.206 [0.110, 0.301]
<20	2503	83.1	105.92 ± 13.83			
20 ~ 24	509	16.9	103.07 ± 13.995			
Grade				3.514	0.015*	0.003 [0.0001, 0.008]
freshman	1035	34.4	106.38 ± 13.06			
sophomore	774	25.7	104.48 ± 14.44			
junior	634	21	105.73 ± 14.38			
senior	569	18.9	104.71 ± 13.98			
Place of origin				−4.5	< 0.01	−0.163 [−0.235, −0.092]
countryside	1390	46.1	104.22 ± 13.28			
cities and towns	1622	53.9	106.48 ± 14.32			
Whether you are an only child				2.08	0.038*	0.084 [0.007, 0.161]
yes	957	31.8	106.23 ± 14.82			
no	2055	68.2	105.07 ± 13.43			
Whether you are a class cadre				3.1	< 0.01	0.131 [0.048, 0.214]
yes	746	24.8	106.8 ± 13.6			
no	2266	75.2	104.99 ± 13.97			

*p < 0.05.

### Correlation analysis

To examine the correlation between perceived social support, psychological capital, stigma for seeking professional psychological help and attitudes toward seeking professional psychological help, a correlation analysis was first conducted on the four variables. The results showed a significant negative correlation between attitudes toward seeking professional psychological help and stigma for seeking professional psychological help (*r* = −0.641, *p* < 0.01), a significant positive correlation with perceived social support and psychological capital (*r* = 0.452, 0.449, *p* < 0.01), and a significant negative correlation between perceived social support and psychological capital and stigma for seeking professional psychological help (*r* = −0.396, – 0.486, *p* < 0.01). The specific results are shown in Table 6, which can be further tested for mediating associations.

**Table 6:** Correlation test.

Variable	Perceived social support	Psychological capital	Stigma for seeking professional psychological help	Attitudes toward seeking professional psychological help
Perceived social support	1			
Psychological capital	0.614**	1		
Stigma for seeking professional psychological help	−0.396**	−0.486**	1	
Attitudes toward seeking professional psychological help	0.452**	0.449**	−0.641**	1

** *p* ＜ 0.01.

### Path coefficient test

**Fig 2:**
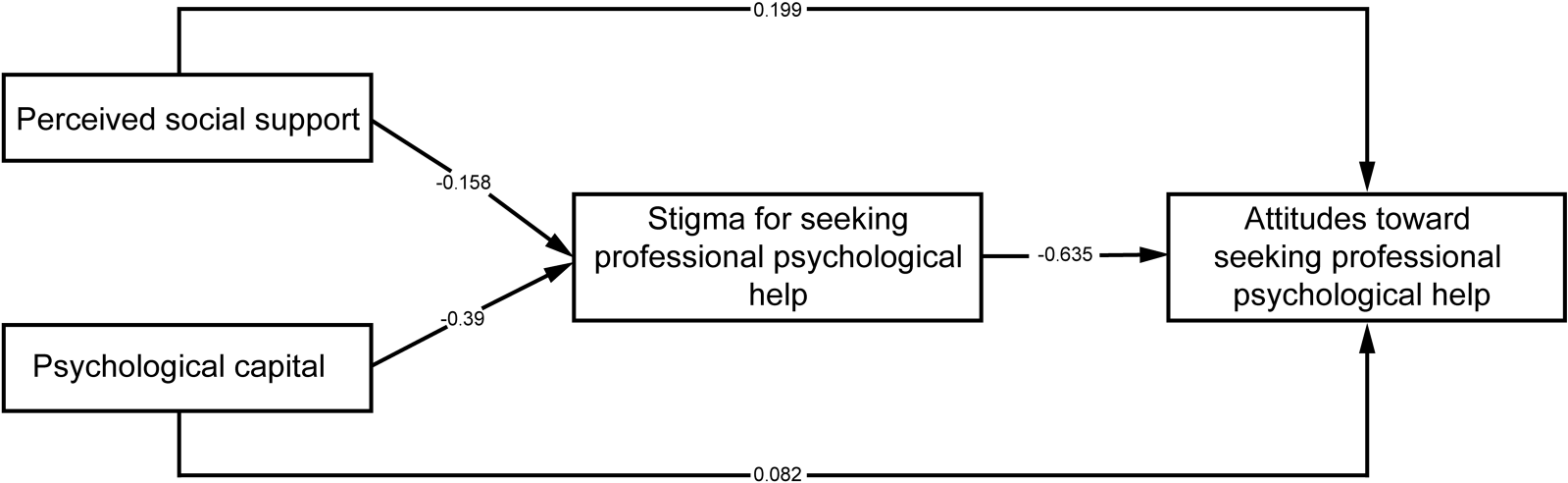
Path coefficient diagram.

The data in this study were standardized to further examine the associations within the model. The results showed that perceived social support and psychological capital were directly and positively associated with attitudes toward seeking professional psychological help (*β* = 0.199, 0.082, *p* < 0.01), supporting H1 and H2. Perceived social support and psychological capital significantly negatively associated with stigma for seeking professional psychological help (*β* = −0.158, −0.39, *p* < 0.01). Stigma for seeking professional psychological help was significant negatively associated with attitudes toward seeking professional psychological help (*β* = −0.635, *p* < 0.01). These paths were all statistically significant. See Table 7 and Fig 2 for more details.

**Table 7:** Path coefficient test.

Paths	Standard estimate	S.E.	C.R.	*p*
Perceived social support→Attitudes toward seeking professional psychological help	0.199	0.02	9.152	< 0.01
Perceived social support→ Stigma for seeking professional psychological help	−0.158	0.004	−5.985	< 0.01
Psychological capital→Attitudes toward seeking professional psychological help	0.082	0.017	3.438	< 0.01
Psychological capital→ Stigma for seeking professional psychological help	−0.39	0.003	−13.332	< 0.01
Stigma for seeking professional psychological help→ Attitudes toward seeking professional psychological help	−0.635	0.173	−24.386	< 0.01

### Mediation analysis

Controlling for gender, age, grade, place of origin, whether or not you are an only child and whether or not you are a class cadre, mediation analysis were further conducted using the Bootstrap method with 5000 samples and 95% confidence intervals were calculated. The results indicated that both the direct association of perceived social support and psychological capital with attitudes toward seeking professional psychological help and the indirect association through stigma for seeking professional psychological help were significant, with 95% confidence intervals excluding 0, suggesting the presence of a mediating effect. The mediating association for stigma for seeking professional psychological help between perceived social support and attitudes toward seeking professional psychological help was 0.1, with a 95% CI of [0.059, 0.138] excluding 0, accounting for 16% of the total association. The mediating association for stigma for seeking professional psychological help between psychological capital and attitudes toward seeking professional psychological help was 0.248, with a 95% CI of [0.21, 0.29] excluding 0, accounting for 39% of the total association, supporting H3 and H4. Stigma for seeking professional psychological help had a stronger mediating association with psychological capital and attitudes toward seeking professional psychological help than with perceived social support and attitudes toward seeking professional psychological help. See Table 8 for details.

**Table 8:** Mediated effects test.

Paths	Effect type	Standardized estimate	SE	Proportion of total effect	95% CI lower	95% CI upper
Perceived social support → Stigma for seeking professional psychological help → Attitudes toward seeking professional psychological help	direct effect_1	0.199	0.024	0.32	0.153	0.245
	indirect effect_1	0.1	0.02	0.16	0.059	0.138
Psychological capital → Stigma for seeking professional psychological help → Attitudes toward seeking professional psychological help	direct effect_2	0.082	0.026	0.13	0.028	0.132
	indirect effect_2	0.248	0.021	0.39	0.21	0.29
total direct effect		0.281	0.023	0.45	0.236	0.327
total indirect effect		0.348	0.017	0.55	0.314	0.382
total effect		0.63	0.019	1	0.59	0.666
indirect effect_1 – indirect effect_2		−0.147	0.036		−0.224	−0.081

## Discussion

### The direct effect of perceived social support on attitudes toward seeking professional psychological help

This study found a significant positive correlation between perceived social support and attitudes toward seeking professional psychological help, which is consistent with previous research findings [58]. This suggests that individuals with higher levels of perceived social support tended to have more rational, objective, and positive attitudes toward seeking psychological help. Perceived social support often originates from significant others in one’s immediate circle, and individuals are frequently associated with an individual’s behavioral attitudes. When individuals perceive support from significant others, they are more likely to align with others’ positive expectations, which may contribute to a more favourable psychological attitude toward seeking help [59]. Individuals situated within a high-perceived social support environment are more likely to confide in trusted individuals regarding daily pressures, emotional fluctuations, and uncertainties [60]. First, this process of confiding may help individuals’ understanding of mental health conditions and seeking psychological support. Second, through open discussions about mental health issues and seeking help, support providers may offer positive responses and constructive feedback, which could help college students reduce their catastrophic and exceptionalized perceptions of mental health conditions and the need for psychological help. This emotional support from significant others not only fulfills individuals’ needs for autonomy and belonging but also helps them perceive professional psychological help as a beneficial and manageable autonomous decision rather than a sign of ‘loss of control’ or ‘incompetence.’ This perception may encourage individuals to seek professional psychological help, and it’s positively related to attitudes toward seeking professional psychological help.

### The direct effect of psychological capital on attitudes toward seeking professional psychological help

This study confirms that psychological capital exhibits a significant positive correlation with attitudes toward seeking professional psychological help, which aligns with existing research [61]. This suggests that college students with higher levels of psychological capital tend to have greater psychological resilience, which may help them recover more quickly from setbacks and resist the tendency to completely negate their self-worth due to temporary emotional fluctuations. Specifically, college students with greater psychological capital are often associated with higher levels of self-efficacy, hopefulness, resilience, and optimism. These traits are positively associated with individuals’ perceptions and behavioral tendencies toward seeking support, which may contribute to more favorable self-evaluations and manifest greater optimism and self-confidence. Thus, they may approach psychological help with an open and composed attitude. When confronted with psychological difficulties, college students with high levels of psychological capital are more likely to view seeking professional help as a growth-oriented challenge and a proactive investment in psychological resources. They maintain confidence in their ability to overcome present obstacles, which may lead to greater initiative and foster positive expectations regarding the outcome of their support-seeking efforts. This perspective may be associated with a more favourable attitude towards seeking professional psychological help.

### The mediating role of stigma for seeking professional psychological help

This study found that a significant negative correlation between the stigma for seeking professional psychological help among college students and their attitudes toward seeking professional psychological help, which aligns with previous research [11]. The public harbors widespread stereotypes about mental illness and those seeking psychological support. To avoid potential reputational damage and interpersonal risks, individuals may adopt psychological defense mechanisms, which could lead to distrust toward professional mental health practitioners and a reluctance to seek specialist psychological assistance [62]. Under the influence of public stigma, college students may lack accurate social cognition about their need for psychological support, with stigmatized social identities possibly reducing their willingness to seek help. Furthermore, individuals experiencing high levels of stigma for seeking professional psychological help may encounter conflicts with their desired self-image, which could lead to self-criticism and self-loathing. This internal emotional conflict exacerbates the formation of self-stigma, which could contribute to more negative attitudes toward seeking professional psychological help.

The findings of this study indicate that the stigma for seeking professional psychological help mediates the association of perceived social support and attitudes toward seeking professional psychological help, (*β* = 0.1, 95% CI [0.059, 0.138]), accounting for 16% of the total effect. Previous research has also confirmed the mediating role of stigma associated with seeking psychological help in professional psychological help [63–64]. Specifically, perceived social support is not only associated with higher levels of attitudes toward seeking professional psychological help but may also be indirectly related to help-seeking attitudes by mitigating stigma. Previous research suggests that perceived social support is negatively associated with negative emotions [65]. On the one hand, individuals with high levels of perceived social support experience greater emotional support and positive feedback, which could contribute to enhanced psychological security and a sense of belonging. This may facilitate the development of positive self-evaluations and builds psychological resources to counteract public stigma. On the other hand, those with low perceived social support are prone to feelings of isolation and rejection, which could reinforce their sense of shame and avoidance of seeking help.

The findings of this study further reveal that stigma for seeking professional psychological help plays a significant mediating role in the association between attitudes toward seeking professional psychological help, (*β* = 0.248, 95% CI [0.21, 0.29]), accounting for 39% of the total effect. Previous studies also support this conclusion [66]. This suggests that psychological capital may be associated with college students’ attitudes toward seeking professional psychological help by potentially reducing stigma associated with seeking psychological support. Psychological capital, as a positive psychological resource, may be associated with mitigating the threat of resource loss associated with the stigma of seeking help, which could be linked to more favorable attitudes toward seeking professional psychological help. College students with higher levels of psychological capital possess richer internal psychological resources, which could potentially help them better regulate the stress arising from the stigma associated with seeking professional psychological help and adopt a more proactive attitude towards seeking professional psychological help. Conversely, those with lower psychological capital tend to have relatively fewer psychological resources. When confronted with the stigma for seeking professional psychological help, they may succumb to self-doubt and feelings of shame, which may contribute to avoidance or passive coping strategies.

### Summary

This study aimed to examine the associations between perceived social support and psychological capital and attitudes toward seeking professional psychological help, as well as the mediating role of stigma for seeking professional psychological help. The results indicated that perceived social support and psychological capital were significantly and directly associated with attitudes toward seeking professional psychological help. Furthermore, stigma for seeking professional psychological help was significantly associated with negative attitudes toward seeking professional psychological help, mediating the relationship between perceived social support and psychological capital and attitudes toward seeking professional psychological help. These findings underscore the importance of implementing interventions and preventive measures aimed at enhancing college students’ perceived social support and psychological capital, while simultaneously reducing the stigma for seeking professional psychological help. By increasing perceived social support and psychological capital, and reducing the stigma for seeking professional psychological help, more positive attitudes toward seeking professional psychological help may be developed, ultimately leading to a positive relationship with mental health.

The findings of this study provide empirical evidence and suggest targeted approaches for future research into intervention strategies that could affect college students’ attitudes toward seeking professional psychological help. Potential strategies may include: First, promoting students’ perceived social support by encouraging them to establish and maintain supportive interpersonal relationships. Second, cultivating psychological capital by fostering psychological resources such as self-efficacy and psychological resilience among college students. Third, reducing public stigma by disseminating knowledge about seeking professional psychological help to the public, which may contribute to creating a more supportive social environment. Finally, promoting an objective perspective on psychological help-seeking, which may help avoid the internalization of negative external messages, reduce self-deprecating evaluations, and lessen the impact of self-stigma.

Overall, the findings underscore the need for a comprehensive approach to address the interplay between college students’ perceived social support, psychological capital, stigma for seeking professional psychological help, and their attitudes toward seeking professional psychological help. By simultaneously addressing these factors, strategies could be developed to encourage positive attitudes toward seeking professional psychological help, which may, in turn, support the overall mental health of this group.

### Research limitations

Although this study presents relevant findings regarding the potential mediating pathways between perceived social support and psychological capital, and college students’ attitudes toward seeking professional psychological help, several limitations remain that should be acknowledged and addressed in future research.

First, this study employed convenience sampling, which may present limitations in sample representativeness. Caution is required when generalizing findings to student populations across different types of institutions. Future research may adopt more structured sampling strategies, such as stratified random sampling by institution type, academic discipline, or year group, or cluster sampling using natural class groups or faculties as units. This would enable a more systematic examination of the stability of variable relationships by comparing differences across distinct subgroups. Such approaches would not only enhance the external validity and generalizability of findings but also provide granular evidence for developing targeted psychological education strategies tailored to diverse student populations.

Second, this study primarily relied on self-report questionnaires for data collection, which may introduce common method bias and multicollinearity issues. Although these were addressed through procedural controls and statistical techniques, the potential impact on relationships between covariates cannot be fully eliminated. While these limitations do not undermine the core findings of this study, future research could enhance robustness by employing longitudinal data collection methods that span multiple time points, diverse sources, and self- and other-assessment approaches.

Third, the cross-sectional design of this study captures the relationships between variables at a single point in time, providing valuable data for understanding the interconnections among various factors. However, the causal direction between variables and their dynamic interactive processes cannot be confirmed using data from a single time point. Subsequent research may employ longitudinal tracking designs or explore cross-lagged models to more clearly delineate the developmental trajectories of each hypothesized pathway over time. This approach could help identify critical windows of influence, providing empirical foundations for designing phased, timely intervention programs.

Finally, this study examines the mediating role of stigma for seeking professional psychological help in the relationship between perceived social support, psychological capital, and attitudes toward seeking professional psychological help, offering insights into a potential explanatory pathway. However, the mechanisms influencing help-seeking attitudes themselves are multifaceted. Future research may build upon this foundation by exploring individuals’ internal cognitive and emotional factors, expanding external social and situational contexts, and comparing individual differences with macro-cultural backgrounds. This could contribute to the development of a more comprehensive and refined network model of mechanisms, providing robust empirical evidence for designing targeted intervention programs to more effectively promote college students’ attitudes toward seeking professional psychological help.

## Supporting information

S1 File Dataset. (XLSX)
